# Investigating the Impact of Protein Supplementation from Different Sources on Advanced Glycation End Products in Older Adults in Singapore Following a Healthy Dietary Pattern

**DOI:** 10.3390/nu18172925

**Published:** 2026-09-07

**Authors:** Marcus Ting, Ian En Kai Mak, Yueying Yao, Amelia Shan Mei Chng, Chin Meng Khoo, Jung Eun Kim

**Affiliations:** 1 Department of Food Science & Technology, National University of Singapore, Singapore 117542, Singapore; marcusting@u.nus.edu (M.T.); ian.mak@u.nus.edu (I.E.K.M.); yueying.yao@u.nus.edu (Y.Y.); ameliachng@nus.edu.sg (A.S.M.C.); 2 Department of Medicine, Yong Loo Lin School of Medicine, National University of Singapore, Singapore 117597, Singapore; mdcsec@nus.edu.sg; 3 Bezos Center for Sustainable Protein, National University of Singapore, Singapore 117542, Singapore

**Keywords:** protein metabolism, advanced glycation end products, aging

## Abstract

**Background/Objectives:** Protein supplementation may elevate advanced glycation end products (AGEs) through higher dietary AGE (dAGE) content and increase amino acid-driven endogenous formation. Protein supplementation from different sources may differentially affect AGE levels, possibly through differences in amino acid composition, digestion and metabolism, but evidence from randomized controlled trials (RCTs) remains limited. This study aimed to examine the effect of protein supplementation from either casein (animal-based) or soy (plant-based) protein isolates on circulating, skin, and dAGE levels in older adults following a healthy dietary pattern (HDP). **Methods:** In this 16-week RCT, 55 older adults (mean age: 66 years old) in Singapore were assigned either to control (no supplementation), casein protein isolate supplementation, or soy protein isolate supplementation. This study was registered at clinicaltrials.gov as NCT05400005. **Results and Discussion:** Overall, there were no alterations in dietary, skin and total circulating AGE levels after protein supplementation when adhering to an HDP such as My Healthy Plate (MHP). However, different sources of protein supplementation resulted in differential changes in specific plasma AGE markers. Two-way ANOVA analysis showed that soy protein supplementation led to a significant increase in plasma N(6)-(1-carboxymethyl)-L-lysine (CML) compared to the other groups (week 0: 0.72 ± 0.10, week 16: 0.80 ± 0.10, *p_int_* = 0.040), while a paired *t*-test showed that casein protein supplementation led to a significant within-group increase in plasma pentosidine (week 0: 0.77 ± 0.06, week 16: 0.80 ± 0.05, *p*-value = 0.045). Other AGEs showed no significant changes. **Conclusions:** Protein supplementation while adhering to MHP did not significantly influence overall circulating and skin AGEs in older adults in Singapore. A differential response was observed only for plasma CML levels with soy protein supplementation, whereas the within-group increase in plasma pentosidine following casein supplementation should be interpreted with caution and requires further investigation.

## 1. Introduction

Higher protein diet (HPD) has gained popularity in recent years for its purported health benefits in increasing/maintaining muscle mass while decreasing fat mass and lowering the risk of cardiometabolic diseases [1,2]. In addition, an HPD is particularly recommended for older adults to prevent or delay age-related disease development, such as sarcopenia and osteoporosis [3,4]. However, it has been reported that consumption of an HPD is associated with higher blood levels of advanced glycation end products (AGEs), potentially due to the relatively high AGE content in some protein-rich foods and increased availability of amino acids in the blood after its consumption, which may contribute to endogenous AGE formation [5].

AGEs are a large group of complex and heterogeneous compounds with highly varied structures [6], and they are obtained from diets or produced in the body via the Maillard reaction between reducing sugars and free amino groups, such as on proteins [7]. It has been documented that higher blood levels of AGEs were associated with a higher risk of cardiovascular diseases (CVDs) [8,9], neurodegenerative diseases [10,11] and type 2 diabetes [12]. Blood N^ε^-(1-carboxymethyl)-L-lysine (CML) level, one of the most commonly investigated AGEs [5], was found to be elevated in patients with type 2 diabetes and coronary heart disease [13] and was associated with all-cause mortality and CVD risk [14]. AGEs can also accumulate in skin, and higher skin AGE levels were also found to be associated with mortality in patients with diabetes, cardiovascular and renal diseases [15]. The relationship between AGEs and higher risk of chronic diseases can be explained by several mechanisms. First, AGEs can alter the structures of essential biological macromolecules via chemical modification of proteins, lipids and nucleic acids, which may cause a loss of their respective functions and collectively contribute to cellular dysfunction and tissue damage [6,16]. When binding to the receptor of AGE (RAGE), AGEs can promote the production of reactive oxygen species and upregulate RAGE [17]. These can activate the nuclear factor-kappa B signaling pathway and induce cellular inflammation [16,18,19].

Dietary strategies such as increasing intake of fruits and vegetables, wholegrains and protein sources like fish, legumes and reduced-fat dairy have been shown to reduce AGE intake from the diet [20]. Dietary recommendations such as Dietary Approaches to Stop Hypertension and Mediterranean diets include the aforementioned food items [21]; thus, following these dietary recommendations might be able to prevent dysregulation in AGE levels with an HPD. A recent randomized controlled trial (RCT) involving obese prediabetic participants showed that consuming black soymilk compared to pork significantly lowered circulating fluorescent AGE levels [22]. This indicates that protein intake from plant sources may have beneficial effects on AGE levels. Furthermore, the effects of protein intake on AGE levels may vary according to protein source; for example, casein and soy proteins differ in their amino acid composition and digestion characteristics. Differences in the digestion and postprandial amino acid kinetics of these proteins may influence amino acid availability and potentially contribute to differences in AGE formation or accumulation [23].

However, there is a scarcity of RCTs investigating the impact of protein supplementation from different sources on AGE levels while adhering to a healthy dietary pattern (HDP). Previous studies on the association between dietary protein intake and blood AGE levels were observational and mainly focused on blood CML levels [24,25,26,27,28]. To the best of our knowledge, there were no studies that holistically investigated different AGE levels including blood, skin and dietary AGE levels. This study examined the effect of protein supplementation from different sources, i.e., animal-/plant-based protein, while adhering to an HDP on AGE levels in the blood, skin and diet in older adults. We hypothesized that protein supplementation would result in higher circulating and skin AGE levels and that different protein sources might result in differential responses.

## 2. Materials and Methods

### 2.1. Participants

A total of 68 older adults in Singapore aged 60–80 were recruited from February 2023 to October 2024 based on the following criteria: not following the My Healthy Plate (MHP) diet, not taking dietary supplements that may affect results, healthy kidney function defined as having an estimated glomerular filtration rate ≥ 60 mL/min/1.73 m^2^ [29], healthy liver function defined as aspartate aminotransferase and alanine aminotransferase levels < 3 times the upper limit of normal (51 U/L for males and 36 U/L for females for both enzymes) [30], no allergy to dairy and soy, no gastrointestinal conditions, not smoking, not drinking more than 2 alcoholic drinks a day, and not on medication except those consistently taking medication for hypertension, lowering cholesterol or type 2 diabetes for more than 5 years before study participation and thus considered in stable condition.

**Figure 1:**
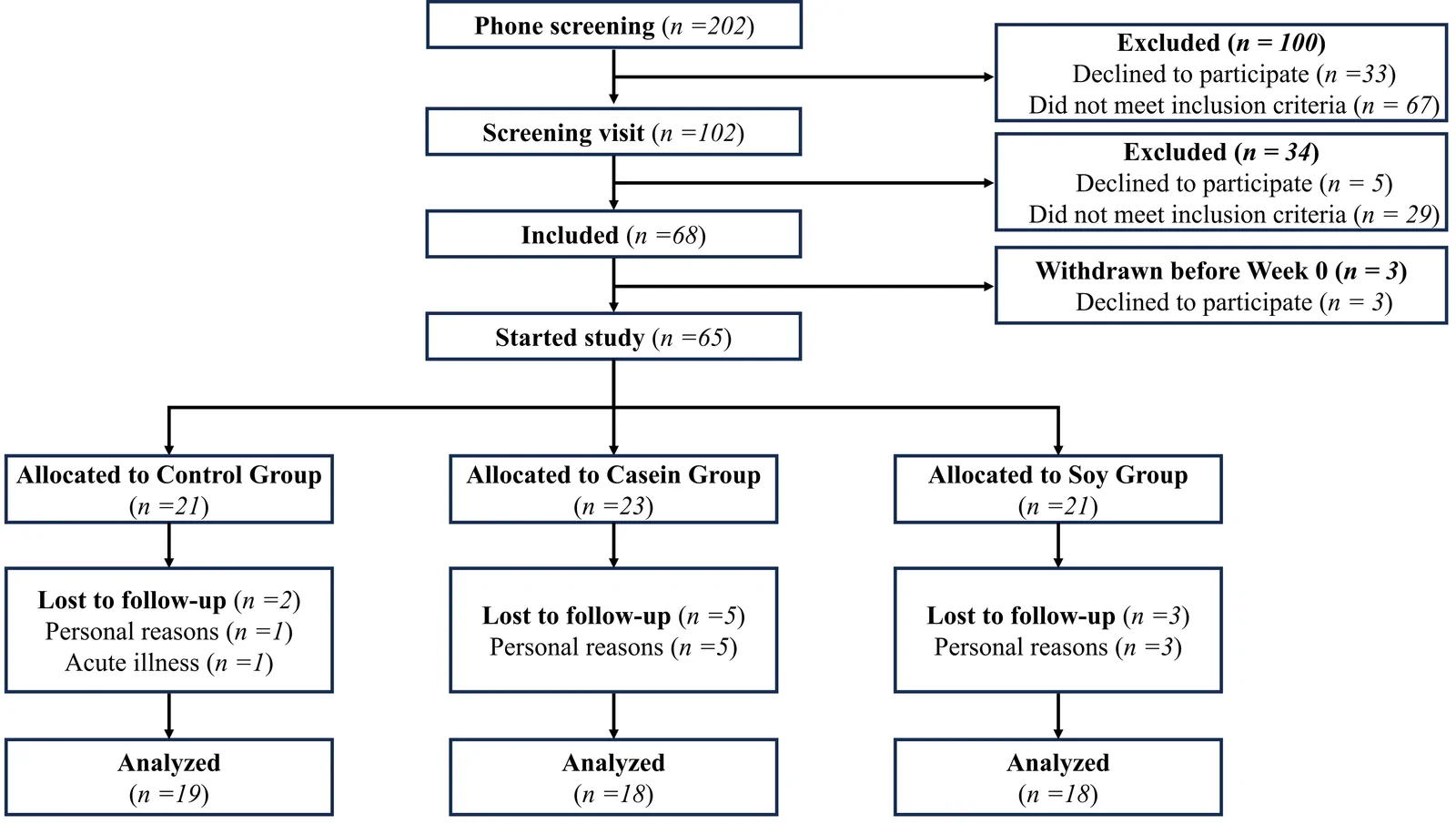
Participant CONSORT flow diagram.

A total of 202 participants were initially contacted for a telephone screening, after which 102 attended an in-person screening at the National University of Singapore Department of Food Science and Technology (Figure 1). Written informed consent was obtained from all participants before any study procedures. We collected information on participants’ medical history, sociodemographic background, lifestyle, and dietary habits. Of the 102 participants screened, 65 eligible participants were randomized to either the control (*n* = 21), casein (*n* = 23) or soy group (*n* = 21). There was a total of 10 participant dropouts: 9 due to personal reasons and 1 due to acute illness not related to the study. None of the participants who withdrew had post-intervention measurements available for AGE analysis. Final analyses were therefore conducted for the per-protocol population of 55 participants who completed the study with adherence (control: *n* = 19; casein: *n* = 18; soy: *n* = 18). The present study was conducted as part of an approved study from NUS IRB (NUS-IRB-2022-316) and was registered at clinicaltrials.gov as NCT05400005 (clinicaltrials.gov/study/NCT5400005) on 6 June 2022. In the original trial protocol, the primary outcomes were sleep-related parameters. Measurements related to AGEs, including plasma CML, CEL, Pen, Pyr, and skin AGE, were pre-specified secondary outcomes and are the focus of the present manuscript. While AGE measurements were pre-specified as secondary outcomes, the specific statistical analyses presented in this manuscript were developed for the secondary analysis after completion of the trial. The primary outcome findings from this trial have not yet been published.

### 2.2. Study Design and Dietary Intervention

**Figure 2:**
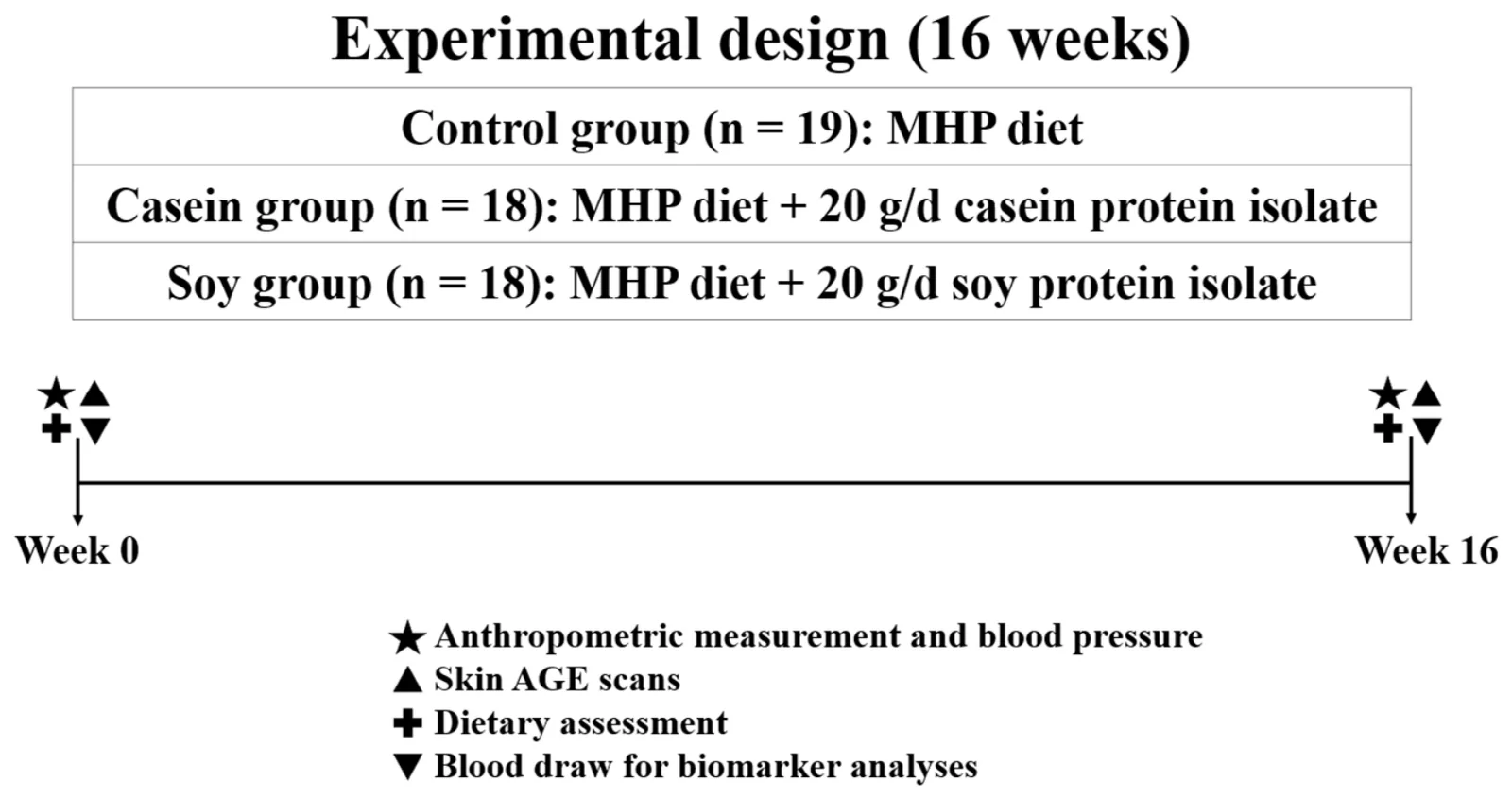
Study design.

This study was a 16-week, single-blinded, three-arm parallel RCT (Figure 2), and eligible participants were randomized into either the control group (MHP diet only), the casein group (MHP diet + 20 g/d casein protein isolate), or the soy group (MHP diet + 20 g/d soy protein isolate). Participants could not be blinded due to the nature of the intervention of receiving no protein supplementation, casein or soy protein supplementation. A research staff member not involved in study enrollment or intervention provision carried out the randomization using Stata/IC 13 (StataCorp, Collage Station, TX, USA) and the allocation of eligible participants to maintain blinding of the main study coordinators. The main study coordinators were not involved in the provision of protein isolates, and participants were instructed not to disclose their allocations to the main study coordinators. Personnel responsible for AGE sample analysis were not provided with information regarding participant allocation. Statistical analyses were also conducted without access to group allocation. All participants followed the MHP diet, while the casein and soy groups were provided with a supplementation of 20 g/d of casein or soy protein isolate, respectively, for 16 weeks. At week 0 and 16, participant anthropometric measurements, blood pressure, dietary intake, skin and blood AGE levels were assessed.

Dietary intervention in the form of MHP and the protein isolate powders was provided in person, with dietary counselling regarding adherence to the MHP by trained researchers. Additionally, an e-booklet detailing the MHP was provided to the participants for easy reference. Briefly, the MHP diet was created by Singapore’s Health Promotion Board, and it comprises three main food groups, namely, wholegrain, meat and others, and fruits and vegetables [31]. MHP recommendations for adults aged 51 years and above include a daily intake of 4–6 servings of wholegrains, 2 servings of fruits and vegetables each, and 3 servings of meat and others, of which at least 1 serving is from dairy or calcium-rich foods.

Protein isolate powders used were unflavored ready-to-mix casein and soy protein isolate powders (Myprotein^©^, Manchester, UK). The powders were packed into identical opaque packets. All participants were instructed to consume 250 mL of UHT low-fat milk daily throughout the intervention. Additionally, participants in the casein and soy groups were instructed to consume 20 g of protein isolate powder with this 250 mL of UHT low-fat milk. Thus, milk intake was standardized across all three groups, while the additional protein source differed between the casein and soy groups. The protein content of the casein and soy protein isolate powders was 16.4 and 18.0 g protein/20 g of powder, respectively. Adherence was ensured by requesting participants to send images of meals consumed via mobile messaging applications, and participants were requested to return empty protein supplementation packets. A compliance rate was estimated using a compliance checklist that participants used to indicate daily whether they adhered to the intervention.

### 2.3. Anthropometric Measurements and Blood Pressure

Height and weight measurements were taken using a stadiometer and digital scale (BSM370, Seoul, Republic of Korea). Waist circumference was measured by a trained study coordinator by taking the measurement using a measuring tape between the lowest rib and the iliac crest. Participants were instructed to take two deep breaths, and the measurement was taken at the second exhalation. All anthropometric measurements were measured twice. Blood pressure was measured using an automatic blood pressure monitor (Omron HEM-7121, Kyoto, Japan). The cuff was placed on the left upper forearm of the participant in a seated position with feet planted on the ground and palm facing upwards. Measurements were collected twice in the form of systolic and diastolic blood pressure.

### 2.4. Dietary Intake Assessment

Participants were instructed to fill in a 3-day food record (3DFR) template provided to them. They were expected to record everything they consumed for 2 weekdays and 1 weekend day in the week leading up to their study visits. They were also instructed to send pictures of their food/meals to ensure compliance and better estimation of food portions. Dietplan7 software (Version 7.00.65, Forestfield Software Ltd., West Sussex, UK) was used for data entry of 3DFR to estimate the nutritional composition of their diets based on the US Department of Agriculture FoodData Central database [32] and Singapore’s Health Promotion Board FOCOS database [33]. The number of servings of MHP food groups was estimated by their 3DFR. UHT low-fat milk consumed as part of the intervention was included in the assessment of dietary protein and dietary AGE intake.

### 2.5. Blood Sample Collection and Analyses

Participants were instructed to fast for at least 10 h before blood collection. Fasting glucose, insulin, glycated hemoglobin, high-density lipoprotein, low-density lipoprotein, total cholesterol and triglyceride concentrations were measured from the fasting blood (Innoquest Diagnostics Pte Ltd., Singapore).

### 2.6. Blood and Skin Advanced Glycation End Product Analyses

We used ultra-performance liquid chromatography–triple quadrupole mass spectrometry (UPLC-TQ-MS/MS) (Waters Xevo TQ-XS, Waters Corporation, Milford, MA, USA) to identify and quantify plasma CML, N^ε^-(1-carboxyethyl)-L-lysine (CEL), pentosidine (Pen) and pyrraline (Pyr) levels. These AGEs were selected because they are among the most extensively characterized AGEs in both foods and human biological samples and are commonly used as biomarkers of dietary AGE exposure and accumulation in the human body. Stock solutions of AGE standards were prepared by dissolving them in ultrapure water and stored at −20 °C before use. Standard solutions for standard curve plotting for the 4 AGEs were prepared in concentrations of 0.01, 0.10, 0.50, and 1.00 μM.

Plasma AGEs were quantified using the same column chemistry, chromatographic elution gradient and mass spectrometric conditions as specified by Xu et al. (2024) in their published paper on their validated method for serum AGE quantification [34]. Xu et al. (2024) [34] reported the intra- and inter-assay coefficient of variation to be <10% across the three days tested; recovery was reported to range from 96.40 to 103.25%; and the limit of detection and quantification for the 20 AGEs tested ranged from 0.10–3.13 ng/mL and 0.5–6.25 ng/mL, respectively. In their validation study, quality-control samples were prepared at low, medium, and high concentrations (10, 50, and 250 ng/mL, respectively) and analyzed alongside calibration solutions. These values represent the published analytical performance characteristics of the underlying validated method and were not independently re-established for the slightly modified procedure used in the present study. Briefly, 200 μL of human plasma was added to 1 mL of methanol and vortexed for 30 s to precipitate the proteins. Centrifugation at 12,000 *g* was performed for 10 min at 4 °C to obtain 1 mL of supernatant. The supernatant was then dried under nitrogen flow and reconstituted with 300 μL of 1% formic acid in ultrapure water. The resulting solution then underwent ultrasound sonication for 10 min before filtering with a 0.22 μm filter and was transferred to an injection vial for UPLC-TQ-MS/MS analyses. This sample preparation procedure primarily captures free plasma AGEs, as protein-bound AGEs were removed during the protein precipitation step and therefore were not quantified in the present analysis.

Liquid chromatography was conducted using an Atlantis C18 AX column (Acquity Premier BEH C18, 1.7 μm, 100 mm × 2.1 mm, Waters Corporation, Milford, MA, USA) at a column temperature of 40 °C. The mobile phases used were 4 mM nonafluoropentanoic acid in water (Solvent A) and 0.1% formic acid in acetonitrile (Solvent B). Gradient elution at a flowrate of 0.3 mL/min was used for CML and CEL, while a flow rate of 0.4 mL/min was utilized for Pen and Pyr as follows: 0.0–1.0 min, 70% Solvent A; 1.0–2.0 min, decrease from 70% to 50% Solvent A; 2.0–3.0 min, decrease from 50% to 10% Solvent A; 3.0–5.0 min, 10% Solvent A; 5.0–5.1 min, increase from 10% to 70% Solvent A; 5.1–6.0 min, re-equilibrated with 70% Solvent A. The injection volume was 10 μL per sample.

Electrospray ionization was conducted using positive mode. Multiple reaction monitoring (MRM) was used to generate suitable MRM transitions for identifying and quantifying AGEs in blood. The capillary voltage used was 3.0 kV, source temperature was 150 °C, desolvation temperature was 450 °C, cone gas flow was 150 L/h, desolvation gas flow was 650 L/h, and nebulizer gas pressure was 7 bar. Optimized parameters and selected MRM functions are listed in Table A1.

Skin AGE levels were assessed using an AGE reader (AGE Reader mu, Diagnoptics Technologies, Groningen, The Netherlands). This non-invasive method uses skin autofluorescence to quantify fluorescent AGEs located in skin tissues and was well correlated with actual skin AGE levels of CML, CEL and Pen [35].

### 2.7. Dietary AGE Analyses

The dAGE levels were estimated from published databases of dAGE levels in various food types [20,36,37,38,39,40,41,42,43,44,45,46,47,48,49,50,51,52,53,54,55,56,57,58,59,60,61,62,63]. CML was expressed as mg CML/kg of food, as this was the most commonly reported form and therefore allowed the greatest number of foods to be matched successfully across the compiled databases. During compilation of the databases, if two or more dAGE values were available for the same food or food type, the following process was adopted to standardize selection: first, the analytical method with greater specificity and accuracy was selected (e.g., liquid chromatography–mass spectrometer methods over enzyme-linked immunosorbent assays); second, if more than one value was available for the same food or food type with similar analytical methodologies, the mean value was calculated to better represent the dAGE value of that food or food type. The dAGE levels were estimated from the 3DFR of the participants, and the most similar food or food type was chosen in the event that the exact match was not available. The cooking method of the food or food type entry was also taken into account to improve the accuracy of dAGE level estimation. The dAGE values for the casein and soy protein isolate powders used in this intervention were also estimated from these existing databases.

### 2.8. Power Calculation and Statistical Analysis

A retrospective power calculation was done using G*Power Version 3.1.9.7 for sample size determination of the present AGE analyses. A previous RCT reported that substituting pork with black soymilk can significantly lower the serum fluorescent AGE intensity (pork: 1600 ± 200; black soymilk: 1400 ± 200, mean ± SD) [22]. Assuming that the present study yields similar results as those seen previously, 17 participants per group will be needed to provide 80% power at α = 0.05 (two-tailed). However, it is important to note that the calculation was based on eight serum fluorescent AGE intensities and not specific to the specific AGEs assessed in the present study.

Rstudio 4.4.1 (Posit Team, Boston, MA, USA) was used for all data and statistical analyses, and results are presented as mean ± SD. One-way ANOVA was used to determine differences in baseline values among the 3 groups. A paired *t*-test with Bonferroni correction was used to analyze the differences between before and after intervention within groups. As only two timepoints were assessed, there was one comparison per treatment group, and the adjusted *p*-values were therefore identical to the unadjusted *p*-values. The chi-squared test was used to determine statistical differences in gender distribution. Repeated measures two-way ANOVA was also used to examine time or time by group interaction effects for all outcomes, coupled with a post hoc Bonferroni test. Log transformation was done for plasma AGE levels to improve the normality of the datasets. A *p*-value of <0.05 was considered statistically significant for all statistical tests.

## 3. Results

### 3.1. Baseline Characteristics

The mean (SD) age was 66 ± 4 years. Participants were all of Chinese ethnicity, with the exception of one Indian participant in the casein group. There were no significant differences in the baseline BMI, blood pressure, lipid-lipoprotein profile and fasting glucose levels between the three groups (Table 1). Based on baseline characteristics, the recruited participants had generally favorable BMI, blood pressure, glucose and lipid-lipoprotein panel levels when compared to reference values provided by the Singapore Heart Foundation and HealthHub Singapore [64,65,66]. Overall, there were no significant time by group effects from week 0 to week 16 for the parameters listed in Table 1.

### 3.2. MHP Servings and Protein Intake

The overall compliance with the interventions had a mean of 93% based on the compliance checklist indicated by the participants. Table 2 shows the MHP servings and protein intake of the participants. Among the three MHP components, there were significant increases in the intake of wholegrains (*p_time_* = 0.003) and meat and others (*p_time_* = 0.006), while no change was observed in fruit and vegetable intakes. Particularly, the casein group showed a significant within-group increase in wholegrain intake (week 0: 0.7 ± 0.8, week 16: 1.1 ± 1.0, *p* = 0.027).

There were no significant changes observed for total protein intake when protein supplementation was excluded. However, after including protein intake from supplementation, there was a time effect when total protein was accounted for in g/d (*p_time_* < 0.001), and this effect was contributed to by a significant increase in total protein intake in the casein and soy groups. When considering total protein in terms of g/kg body weight/d, a cross-sectional study showed that each 0.1 g/kg body weight/d increase in total protein intake was associated with an approximate increase of 13.3 ng/mL in serum CML [25].

**Table 2:** MHP servings and protein intake of participants at week 0 and 16.

Parameters	Control (*n* = 19)		Casein (*n* = 18)		Soy (*n* = 18)		*p*-Value	
	Week 0	Week 16	Week 0	Week 16	Week 0	Week 16	Time	Time × Group
Wholegrains (servings/d)	1.0 ± 0.8	1.4 ± 1.3	0.7 ± 0.8	1.1 ± 1.0 *	1.0 ± 0.8	1.5 ± 0.9	0.003	0.943
Meat and others (servings/d)	2.7 ± 0.8	3.2 ± 1.1	2.7 ± 0.6	2.9 ± 0.8	2.2 ± 0.8	2.8 ± 1.0	0.006	0.534
Fruits & vegetables (servings/d)	3.0 ± 1.5	3.4 ± 1.4	3.6 ± 2.0	3.3 ± 1.6	3.3 ± 1.2	3.0 ± 1.4	0.654	0.300
Total protein ^a^ (g/d)	79 ± 20	87 ± 27	78 ± 16	79 ± 17	73 ± 18	78 ± 24	0.089	0.633
Total protein ^b^ (g/d)	79 ± 20	87 ± 27	78 ± 16	96 ± 17 *	73 ± 18	96 ± 24 *	<0.001	0.094
Total protein ^a^ (g/kg bw/d)	1.3 ± 0.3	1.5 ± 0.5	1.4 ± 0.3	1.4 ± 0.4	1.2 ± 0.3	1.3 ± 0.4	0.117	0.587
Total protein ^b^ (g/kg bw/d)	1.3 ± 0.3	1.5 ± 0.5	1.4 ± 0.3	1.7 ± 0.4	1.2 ± 0.3	1.7 ± 0.4	0.088	0.665

Values are means ± SD. Participants in the casein and soy groups consumed 20 g/day of their respective protein supplementation, providing approximately 16.4 g and 18.0 g of protein, respectively. The powder was consumed with 250 mL of UHT low-fat milk, providing an additional ~8 g of protein, and this amount of milk was consumed daily by all groups. ^a^ Total protein intake excluding protein intake from supplementation. ^b^ Total protein intake including protein intake from supplementation. * Indicates significant difference in the group’s pre- and post-intervention mean values using paired *t*-test analyses at a *p*-value < 0.05.

### 3.3. Dietary, Plasma and Skin AGE Levels

The two-way ANOVA analyses showed no time and time by group interaction effects for both dAGE levels before and after accounting for dAGE content in the protein isolate powders (Table 3).

**Figure 3:**
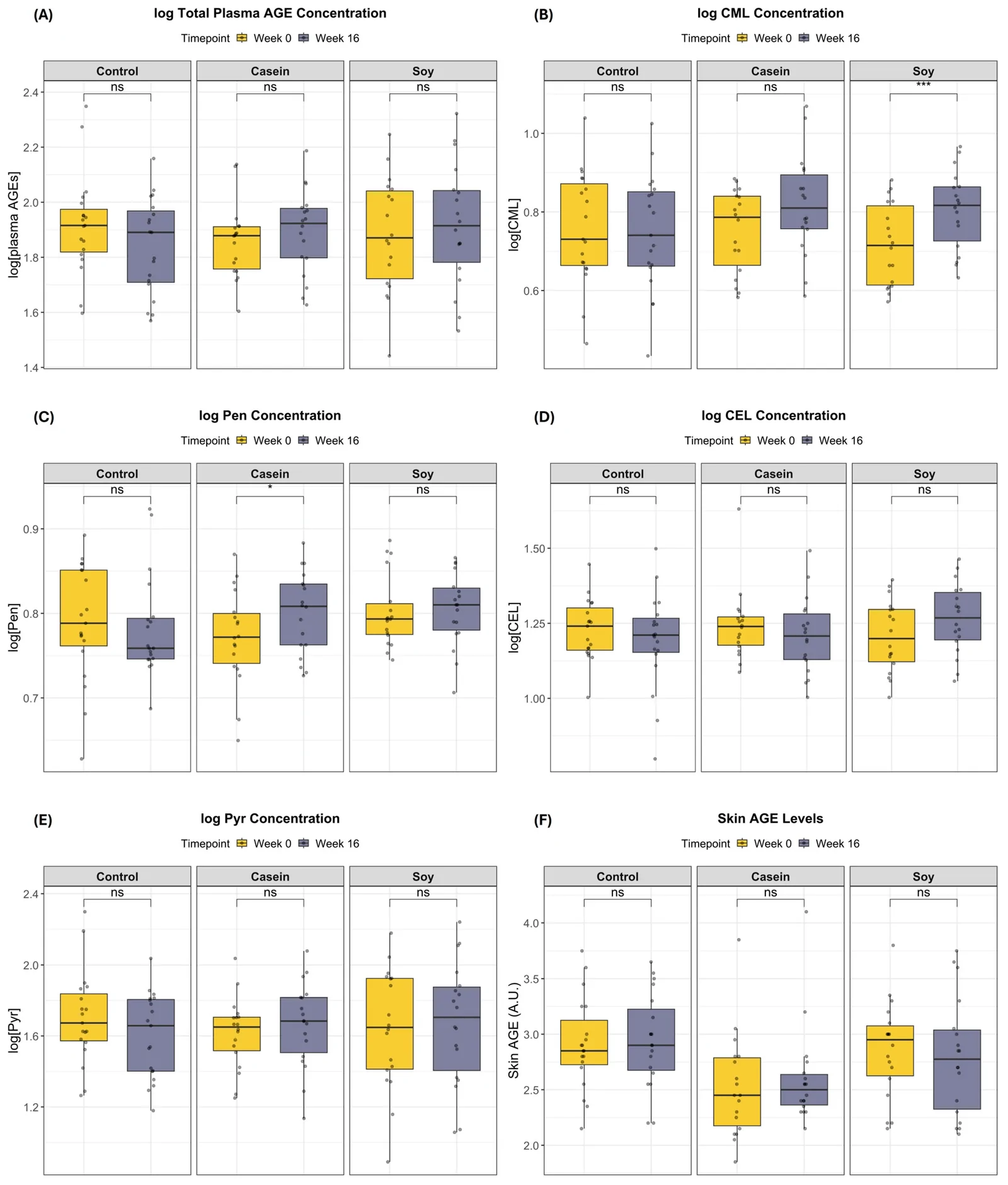
Within-group changes in log-scale concentrations of total plasma AGE levels (**A**), CML (**B**), Pen (**C**), CEL (**D**), Pyr (**E**) and skin AGE levels (**F**) from week 0 to 16 for control, casein and soy groups. Paired *t*-test conducted with significance determined at *p*-value < 0.05, *: *p* < 0.05, ***: *p* < 0.001; ns: not significant.

Within-group changes in plasma and skin AGE levels are summarized in Figure 3, and the two-way ANOVA analyses are presented in Table A2. Total plasma AGE levels were not significantly altered after 16 weeks within the groups (Figure 3A). The two-way ANOVA analyses indicated a significant time by group interaction effect (*p_int_* = 0.040) (Table A2) for plasma CML levels, and this effect was particularly attributed to a significant within-group increase in the soy group (week 0: 0.72 ± 0.10, week 16: 0.80 ± 0.10, *p*-value < 0.001) (Figure 3B). No significant time and time by group interaction effects were observed for total plasma AGE (*p_int_* = 0.195), CEL (*p_int_* = 0.098), Pen (*p_int_* = 0.220), Pyr (*p_int_* = 0.303) or skin AGE (*p_int_* = 0.286) levels (Table A2). However, the casein group showed a significant within-group increase in Pen levels (week 0: 0.77 ± 0.06, week 16: 0.80 ± 0.05, *p*-value = 0.045) (Figure 3C). No significant within-group changes in other specific plasma AGEs and skin AGE levels were detected in any group (Figure 3D–F).

## 4. Discussion

Although high levels of AGEs have been associated with higher dietary protein intake and AGEs are also associated with various cardiometabolic diseases [25,67,68], few studies have directly examined the impact of protein supplementation from different sources on circulating or tissue AGE levels. In this study, we found that protein supplementation does not alter levels of circulating total AGEs in blood and accumulated AGEs under the skin when individuals adhered to a MHP diet. However, different protein sources resulted in differential responses in specific AGEs. The soy group showed a significant increase in plasma CML levels compared to the other groups, while plasma Pen levels were significantly increased within the casein group.

### 4.1. Impact of Protein Supplementation on dAGE and Total Plasma AGE Levels

Although there was an increase in total protein intake with supplementation, we did not observe an increase in dAGE intake. This could be partly because estimated dAGE only reflects CML content and dAGE contribution from the supplemented protein isolates was relatively small, estimated at 1.02 mg and 1.48 mg of CML in 20 g of casein and soy protein isolate, respectively. There was also no increase in total plasma AGE levels, and this could be possible due to characteristics of the MHP dietary pattern, including its emphasis on nutrient-dense foods like wholegrains. In a previous 4-week RCT involving 51 healthy adults, consumption of a high wholegrain diet could be maintained, whereas a high meat protein diet increased plasma CEL levels [69]. Wholegrains are commonly rich in dietary fiber, and a cross-sectional study of 128 patients on hemodialysis showed that higher dietary fiber intake was associated with significantly lower serum AGE levels [70]. AGE formation was shown to be driven by hyperglycemia and subsequent inflammatory conditions due to increased reactive oxygen species production [71]. Wholegrains can delay gastric emptying and reduce the rate of digestion and absorption of glucose, thus reducing blood glucose levels [72,73]. Wholegrains also contain a high antioxidant content, and by increasing the overall antioxidant capacity of the body, they can reduce free radicals and systemic inflammation [72]. Although wholegrain consumption has previously been associated with lower AGE exposure and improved metabolic health, the modest increase observed in the present study was unlikely to be sufficient to explain the observed AGE outcomes. Therefore, the contribution of wholegrain intake should be interpreted cautiously.

### 4.2. Impact of Protein Supplementation on Individual AGE Levels

When considering specific AGE levels, soy protein supplementation increased plasma CML levels with a significant time by group interaction, and this finding agrees with the Health, Aging, and Body Composition (Health ABC) cohort study, which reported higher serum CML levels with higher protein intake [25]. However, the Health ABC study indicated that higher intakes of both animal and vegetable protein were associated with higher blood CML levels, whereas our study only found a significant increase in plasma CML levels, which was mainly driven by soy protein supplementation, as seen by post hoc analysis. One possible explanation for this observation may relate to differences in amino acid composition between soy and casein proteins, including that the relatively high lysine residues in soy protein compared to milk proteins might result in a higher plasma CML level observed here [74].

In contrast, a significant within-group increase in Pen was observed following casein supplementation, albeit with caution in interpretation, as the time by group interaction was not statistically significant. One possible explanation may potentially be related to the digestion characteristics of casein. Casein is generally considered a slowly digested protein and has a relatively long gastrointestinal residence time, resulting in a more sustained release of amino acids [75]. This prolonged availability of amino acids may potentially influence endogenous AGE formation via crosslinking between lysine and arginine from casein proteins with reactive carbonyl species, like 3-deoxyglucosone, and fructose present in the diet, which increases the formation of Pen in the gastrointestinal tract [75,76]. However, as postprandial amino acid concentrations and AGE formation pathways were not directly measured in the present study, these explanations remain speculative.

While modest, the observed increase in plasma CML levels following soy protein supplementation and the within-group increase in plasma Pen levels provided insights into the differential effects of protein supplementation on AGE levels and warrant further investigation. The observed CML response in the soy group may potentially relate to differences in amino acid composition, while the within-group changes in Pen levels of the casein group provide a hypothesis for further investigation into the role of protein metabolism in AGE formation. These potential mechanisms were not directly assessed in the present study and require confirmation in future mechanistic studies.

### 4.3. Impact of Protein Supplementation on Skin AGE Levels

In line with there being no changes observed in total plasma AGE levels, skin AGE levels were not significantly altered after protein supplementation, which can also be partly attributed to the increase in wholegrain intake. Similarly, a previous study found that higher cereal consumption was inversely related to skin AGE levels in a cohort of healthy middle-aged adults [76]. It has been suggested that higher intake of wholegrains could reduce the formation of AGEs in skin due to increased antioxidant capacity [77]. However, caution is warranted when interpreting this finding, as wholegrain intake was not experimentally isolated in this study and no significant time by group interaction was observed; thus, its contribution to skin AGE levels remains speculative. Additionally, skin AGE accumulation is generally a slower process than plasma AGE turnover. For example, collagen is a long-lived protein in the skin, with its half-life estimated to be about 10–15 years. Consequently, the accumulation of skin AGEs might reflect skin AGE burden long before the start of this intervention, and the magnitude of any intervention-related change may have been insufficient to produce a measurable difference against the pre-existing skin AGE burden within 16 weeks [78,79]. This may explain the lack of change in skin AGE levels; hence, a longer intervention period may be required to capture significant changes in skin AGE values. Another possible reason is the presence of non-AGE fluorophores such as tyrosine and melanin, which can contribute to background fluorescence and obscure any small changes in skin autofluorescence values contributed by AGE fluorophores [79,80]. Lastly, it is important to note that the measurement of skin AGEs is based on skin autofluorescence, which primarily reflects the accumulation of fluorescent AGEs under the skin [81]. Therefore, caution is warranted when interpreting skin AGE values.

### 4.4. Strengths and Limitations

There are several strengths to this study. To our knowledge, this is the first RCT to investigate the effects of protein supplementation with different protein sources on different AGE levels while adhering to an HDP among older adults. Particularly, the inclusion of both soy and casein protein isolates allowed for a direct comparison between animal- and plant-based protein sources without the need to account for other nutrients that are higher in protein-rich foods. We used a holistic approach in assessing plasma, skin and dAGE levels to provide an integrated perspective of AGE exposure and accumulation. The majority of previous studies measured CML only. However, there are several limitations. First, we only quantified free AGEs, and since protein-bound AGEs were mostly removed during the protein precipitation step of blood sample preparation, the current results might not have captured the changes in protein-bound AGEs. For example, 75% of the Pen in coffee was in free form, while almost all the Pen in pretzel sticks was protein-bound [82]. For this reason, proper AGE kinetics studies in the human body for specific AGEs and the forms they exist in are required for a more in-depth understanding of AGE absorption and metabolism. Second, dAGE is estimated only as CML, and this cannot fully capture the entire dAGE intake profile of their diets and the protein supplementations. Furthermore, there is a scarcity of Asian cuisines in existing databases for dAGE, which could not completely capture the Singaporean dAGE intake despite best efforts for standardization in our paper. In addition, cooking methods, which greatly impact the dAGE content of foods, were not controlled for in this study. Third, despite AGE levels being associated with disease development, the clinical significance of the observed AGE level changes in our study remains unclear, as clinical thresholds or supposed healthy ranges for AGE levels have yet to be established. Fourth, the predominantly Chinese composition of the study population, while unintended, may limit the generalizability of the findings to other ethnic groups in Singapore. Additionally, the sample size of the study might be modest; thus, future larger studies should be conducted. Fifth, supplementation with a protein isolate does not completely represent dietary protein intake due to the absence of food matrix effects and is not commonly eaten as part of a meal. Furthermore, casein and soy do not fully represent animal or plant protein intake as a whole, especially since UHT low-fat milk was instructed to be consumed together with the protein supplementation. Despite no significant time by group interaction effect in total protein intake, including the supplemented protein content, it is important to note that there was a difference of ~1.6 g in the actual protein content provided by the casein and soy powders even though both powders were matched at 20 g/day. Although minimal, this difference should nevertheless be considered when interpreting comparisons between the casein and soy groups. Lastly, since the AGE outcomes were pre-specified secondary outcomes, the statistical analyses were developed after trial completion. Moreover, the study was not specifically powered to detect time by group interactions for individual AGE biomarkers. Furthermore, multiple AGE outcomes were examined, which may increase the risk of type I error despite adjustment of the post hoc timepoint comparisons. In addition, analyses were done per-protocol instead of intention-to-treat, and participants could not be blinded due to the nature of the intervention.

## 5. Conclusions

In conclusion, this study demonstrated that with adherence to the MHP diet, protein supplementation did not significantly influence total circulating AGE and skin AGE levels in older adults in Singapore. However, soy protein supplementation did elicit a differential response in plasma CML levels, whereas the within-group changes in plasma Pen levels warrants further investigation. These findings provide preliminary evidence that protein sources may differentially influence specific AGE biomarkers, especially plasma CML, and supports the need for further adequately powered and longer interventional trials to investigate whether different protein sources regulate AGE responses when consumed as part of an HDP.

## Figures and Tables

**Table 1:** Baseline characteristics of participants.

Parameters ^1^	Control (*n* = 19)	Casein (*n* = 18)	Soy (*n* = 18)	*p*-Value
Gender (M/F, *n*)	11/8	7/11	9/9	0.511
BMI (kg/m^2^)	23.2 ± 2.6	23.1 ± 3.0	23.4 ± 3.5	0.947
SBP (mmHg)	122 ± 15	125 ± 17	127 ± 16	0.703
DBP (mmHg)	76 ± 8	75 ± 9	77 ± 12	0.702
TC (mg/dL)	221 ± 31	214 ± 38	213 ± 36	0.710
HDL (mg/dL)	66 ± 17	68 ± 19	69 ± 15	0.908
LDL (mg/dL)	135 ± 23	127 ± 30	126 ± 28	0.534
TG (mg/dL)	101 ± 36	98 ± 50	89 ± 30	0.624
Fasting glucose (mg/dL)	86 ± 11	91 ± 15	86 ± 9	0.329

^1^ BMI: body mass index, SBP: systolic blood pressure, DBP: diastolic blood pressure, TC: total cholesterol, HDL: high-density lipoprotein, LDL: low-density lipoprotein, TG: triglyceride. Values are means ± SD.

**Table 3:** Overview of changes in dAGE levels after 16 weeks.

dAGE (mg CML/Day)	Control (*n* = 19)		Casein (*n* = 18)		Soy (*n* = 18)		*p*-Value	
	Week 0	Week 16	Week 0	Week 16	Week 0	Week 16	Time	Time × Group
dAGE ^a^	9.55 ± 4.92	7.58 ± 3.02	8.92 ± 13.3	8.13 ± 6.16	8.04 ± 3.77	8.30 ± 5.16	0.434	0.687
dAGE ^b^	9.55 ± 4.92	7.58 ± 3.02	8.92 ± 13.3	9.15 ± 6.16	8.04 ± 3.77	9.78 ± 5.16	0998	0.354

^a^ dAGE levels not accounting for dAGE content of the protein isolates used in the intervention. ^b^ dAGE levels accounting for dAGE content of the protein isolates used in the interventions.

## Data Availability

The data presented in this study will be made available on request from the corresponding author. The data are not publicly available due to restrictions on informed consent and institutional guidelines.

## Abbreviations

The following abbreviations are used in this manuscript:

3DFR	3-day food record
AGEs	Advanced glycation end products
BMI	Body mass index
CEL	N^ε^-(1-carboxyethyl)-L-lysine
CML	N^ε^-(1-carboxymethyl)-L-lysine
CVD	Cardiovascular diseases
dAGE	Dietary AGE
DBP	Diastolic blood pressure
HDL	High-density lipoprotein
Health ABC	Health, Aging and Body Composition
HDP	Healthy dietary pattern
HPD	Higher protein diet
LDL	Low-density lipoprotein
MHP	My Healthy Plate
Pen	Pentosidine
Pyr	Pyrraline
RAGE	Receptor of AGE
RCT	Randomized controlled trial
SBP	Systolic blood pressure
TC	Total cholesterol
TG	Triglyceride
UPLC-TQ-MS/MS	Ultra-performance liquid chromatography–triple quadrupole mass spectrometry

## Appendix A

**Table A1:** MRM functions of different blood AGE analytes for detection.

Analyte	Precursor (*m*/*z*)	Product (*m*/*z*)	Cone Voltage (V)	Collision Energy (V)
CML	204.94	83.92	10	19
		130.01	10	12
CEL	218.92	83.98	13	18
		130.06	13	13
Pen	379.14	134.81	13	38
		186.86	13	40
Pyr	254.94	148.14	8	18
		174.92	8	12

CML: N^ε^-(1-carboxymethyl)-L-lysine, CEL: N^ε^-(1-carboxyethyl)-L-lysine, Pen: pentosidine, Pyr: pyrraline.

**Table A2:** Overview of changes in plasma and skin AGE levels after 16 weeks.

AGE Levels	Control (*n* = 19)		Casein (*n* = 19)		Soy (*n* = 19)		*p*-Value	
	Week 0	Week 16	Week 0	Week 16	Week 0	Week 16	Time	Time × Group
Total plasma AGEs	1.92 ± 0.18	1.84 ± 0.18	1.86 ± 0.13	1.89 ± 0.15	1.88 ± 0.21	1.92 ± 0.22	0.931	0.195
CML	0.78 ± 0.14	0.75 ± 0.15	0.75 ± 0.11	0.82 ± 0.13	0.72 ± 0.10	0.80 ± 0.10 *	0.004	0.040
CEL	1.23 ± 0.10	1.19 ± 0.16	1.24 ± 0.12	1.21 ± 0.13	1.21 ± 0.12	1.27 ± 0.12	0.867	0.098
Pen	0.79 ± 0.07	0.78 ± 0.06	0.77 ± 0.06	0.80 ± 0.05 *	0.80 ± 0.04	0.81 ± 0.04	0.327	0.220
Pyr	1.70 ± 0.26	1.60 ± 0.24	1.62 ± 0.20	1.66 ± 0.24	1.64 ± 0.34	1.67 ± 0.35	0.866	0.303
Skin AGE (A.U.)	2.91 ± 0.41	2.94 ± 0.42	2.53 ± 0.47	2.60 ± 0.44	2.86 ± 0.44	2.79 ± 0.53	0.702	0.286

CML: N^ε^-(1-carboxymethyl)-L-lysine, CEL: N^ε^-(1-carboxyethyl)-L-lysine, Pen: pentosidine, Pyr: pyrraline. Values are means ± SD. Two-way ANOVA was conducted with significance determined at a *p*-value < 0.05. Units for blood AGEs are in log nM; skin AGE levels are in arbitrary units; and dAGE levels are in mg CML/day). * Indicates significant differences after paired *t*-test analyses for the group’s pre- and post-intervention mean values at a *p*-value < 0.05.

## References

[1] Evangelista L.S., Jose M.M., Sallam H., Serag H., Golovko G., Khanipov K., Hamilton M.A., Fonarow G.C. (2021). High-protein vs. standard-protein diets in overweight and obese patients with heart failure and diabetes mellitus: Findings of the Pro-HEART trial. ESC Heart Fail..

[2] Layman D.K., Clifton P., Gannon M.C., Krauss R.M., Nuttall F.Q. (2008). Protein in optimal health: Heart disease and type 2 diabetes. Am. J. Clin. Nutr..

[3] Coelho-Junior H.J., Calvani R., Azzolino D., Picca A., Tosato M., Landi F., Cesari M., Marzetti E. (2022). Protein intake and sarcopenia in older adults: A systematic review and meta-analysis. Int. J. Environ. Res. Public Health.

[4] Nowson C., O’Connell S. (2015). Protein requirements and recommendations for older people: A review. Nutrients.

[5] Uribarri J., Tuttle K.R. (2006). Advanced glycation end products and nephrotoxicity of high-protein diets. Clin. J. Am. Soc. Nephrol..

[6] Schmitt A., Schmitt J., Münch G., Gasic-Milencovic J. (2005). Characterization of advanced glycation end products for biochemical studies: Side chain modifications and fluorescence characteristics. Anal. Biochem..

[7] Sergi D., Boulestin H., Campbell F.M., Williams L.M. (2021). The role of dietary advanced glycation end products in metabolic dysfunction. Mol. Nutr. Food Res..

[8] Fishman S.L., Sonmez H., Basman C., Singh V., Poretsky L. (2018). The role of advanced glycation end-products in the development of coronary artery disease in patients with and without diabetes mellitus: A review. Mol. Med..

[9] Hartog J.W., Voors A.A., Bakker S.J., Smit A.J., van Veldhuisen D.J. (2007). Advanced glycation end-products (AGEs) and heart failure: Pathophysiology and clinical implications. Eur. J. Heart Fail..

[10] Li J., Liu D., Sun L., Lu Y., Zhang Z. (2012). Advanced glycation end products and neurodegenerative diseases: Mechanisms and perspective. J. Neurol. Sci..

[11] Sasaki N., Fukatsu R., Tsuzuki K., Hayashi Y., Yoshida T., Fujii N., Koike T., Wakayama I., Yanagihara R., Garruto R. (1998). Advanced Glycation End Products in Alzheimer’s Disease and Other Neurodegenerative Diseases. Am. J. Pathol..

[12] Yan S.F., Ramasamy R., Schmidt A.M. (2008). Mechanisms of disease: Advanced glycation end-products and their receptor in inflammation and diabetes complications. Nat. Clin. Pract. Endocrinol. Metab..

[13] Kilhovd B.K., Berg T.J., Birkeland K.I., Thorsby P., Hanssen K.F. (1999). Serum levels of advanced glycation end products are increased in patients with type 2 diabetes and coronary heart disease. Diabetes Care.

[14] Semba R.D., Bandinelli S., Sun K., Guralnik J.M., Ferrucci L. (2009). Plasma carboxymethyl-lysine, an advanced glycation end product, and all-cause and cardiovascular disease mortality in older community-dwelling adults. J. Am. Geriatr. Soc..

[15] Cavero-Redondo I., Soriano-Cano A., Álvarez-Bueno C., Cunha P.G., Martínez-Hortelano J.A., Garrido-Miguel M., Berlanga-Macías C., Martínez-Vizcaíno V. (2018). Skin Autofluorescence–Indicated Advanced Glycation End Products as Predictors of Cardiovascular and All-Cause Mortality in High-Risk Subjects: A Systematic Review and Meta-analysis. J. Am. Heart Assoc..

[16] Heidland A., Sebekova K., Schinzel R. (2001). Advanced glycation end products and the progressive course of renal disease. Am. J. Kidney Dis..

[17] Schmidt A.M., Clynes R., Moser B., Yan S.F., Ramasamy R., Herold K. (2007). Receptor for AGE (RAGE): Weaving tangled webs within the inflammatory response. Curr. Mol. Med..

[18] Xie J., Méndez J.D., Méndez-Valenzuela V., Aguilar-Hernández M.M. (2013). Cellular signalling of the receptor for advanced glycation end products (RAGE). Cell. Signal..

[19] Wrobel K., Wrobel K., Ortiz S.J., Escobosa A.R.C. (2017). What are AGEs, their chemical structure, and how can they be measured?. Dietary AGEs and Their Role in Health and Disease.

[20] Uribarri J., Woodruff S., Goodman S., Cai W., Chen X., Pyzik R., Yong A., Striker G.E., Vlassara H. (2010). Advanced glycation end products in foods and a practical guide to their reduction in the diet. J. Am. Diet. Assoc..

[21] Moorthi R.N., Vorland C.J., Gallant K.M.H. (2017). Diet and diabetic kidney disease: Plant versus animal protein. Curr. Diabetes Rep..

[22] Chang Y.-H., Lee P.-N., Chen C.-H., Yang H.-Y., Wu C.-H., Doong J.-Y., Yeh W.-J. (2025). Substituting animal protein with black soymilk reduces advanced glycation end product level and improves gut microbiota composition in obese prediabetic individuals: A randomized crossover intervention trial. Food Funct..

[23] Bos C., Metges C.C., Gaudichon C., Petzke K.J., Pueyo M.E., Morens C., Everwand J., Benamouzig R., Tome D. (2003). Postprandial kinetics of dietary amino acids are the main determinant of their metabolism after soy or milk protein ingestion in humans. J. Nutr..

[24] Šebeková K., Krajčovičová-Kudláčková M., Schinzel R., Faist V., Klvanová J., Heidland A. (2001). Plasma levels of advanced glycation end products in healthy, long-term vegetarians and subjects on a western mixed diet. Eur. J. Nutr..

[25] Brinkley T.E., Semba R.D., Kritchevsky S.B., Houston D.K. (2020). Health, Aging, and Body Composition Study. Dietary protein intake and circulating advanced glycation end product/receptor for advanced glycation end product concentrations in the Health, Aging, and Body Composition Study. Am. J. Clin. Nutr..

[26] Chen J., Waqas K., Tan R.C., Voortman T., Ikram M.A., Nijsten T.E., De Groot L.C., Uitterlinden A.G., Zillikens M.C. (2020). The association between dietary and skin advanced glycation end products: The Rotterdam Study. Am. J. Clin. Nutr..

[27] Foroumandi E., Kheirouri S., Nosrati R., Ghodsi R. (2021). Association of dietary intake, medication and anthropometric indices with serum levels of advanced glycation end products, caspase-3, and matrix metalloproteinase-9 in diabetic patients. J. Diabetes Metab. Disord..

[28] Almajwal A.M., Alam I., Abulmeaty M., Razak S., Pawelec G., Alam W. (2020). Intake of dietary advanced glycation end products influences inflammatory markers, immune phenotypes, and antiradical capacity of healthy elderly in a little-studied population. Food Sci. Nutr..

[29] Levey A.S., Eckardt K.-U., Tsukamoto Y., Levin A., Coresh J., Rossert J., Zeeuw D.D., Hostetter T.H., Lameire N., Eknoyan G. (2005). Definition and classification of chronic kidney disease: A position statement from Kidney Disease: Improving Global Outcomes (KDIGO). Kidney Int..

[30] Devarbhavi H., Aithal G., Treeprasertsuk S., Takikawa H., Mao Y., Shasthry S.M., Hamid S., Tan S.S., Philips C.A., George J. (2021). Drug-induced liver injury: Asia Pacific Association of Study of Liver consensus guidelines. Hepatol. Int..

[31] Health Promotion Board My Healthy Plate. https://www.healthhub.sg/programmes/nutrition-hub/eat-more.

[32] U.S. Department of Agriculture, Agricultural Research Service (2019). FoodData Central. https://fdc.nal.usda.gov/.

[33] Health Promotion Board (2023). Energy & Nutrient Composition of Food. https://focos.hpb.gov.sg/eservices/ENCF/.

[34] Xu Y., Huang M., Chen Y., Yu L., Wu M., Kang S., Lin Q., Zhang Q., Han L., Lin H. (2024). Development of simultaneous quantitation method for 20 free advanced glycation end products using UPLC–MS/MS and clinical application in kidney injury. J. Pharm. Biomed. Anal..

[35] Meerwaldt R., Links T., Graaff R., Thorpe S.R., Baynes J.W., Hartog J., Gans R., Smit A. (2005). Simple noninvasive measurement of skin autofluorescence. Ann. N. Y. Acad. Sci..

[36] Fenaille F., Parisod V., Visani P., Populaire S., Tabet J.-C., Guy P.A. (2006). Modifications of milk constituents during processing: A preliminary benchmarking study. Int. Dairy J..

[37] Hull G.L., Woodside J.V., Ames J.M., Cuskelly G.J. (2012). *N*^ε^-(carboxymethyl)lysine content of foods commonly consumed in a Western style diet. Food Chem..

[38] Degen J., Hellwig M., Henle T. (2012). 1,2-dicarbonyl compounds in commonly consumed foods. J. Agric. Food Chem..

[39] Hellwig M., Kiessling M., Rother S., Henle T. (2016). Quantification of the glycation compound 6-(3-hydroxy-4-oxo-2-methyl-4(1*H*)-pyridin-1-yl)-l-norleucine (maltosine) in model systems and food samples. Eur. Food Res. Technol..

[40] Hellwig M., Witte S., Henle T. (2016). Free and Protein-Bound Maillard Reaction Products in Beer: Method Development and a Survey of Different Beer Types. J. Agric. Food Chem..

[41] Hellwig M., Kühn L., Henle T. (2018). Individual Maillard reaction products as indicators of heat treatment of pasta—A survey of commercial products. J. Food Compos. Anal..

[42] Assar S.H., Moloney C., Lima M., Magee R., Ames J.M. (2009). Determination of *N*^ε^-(carboxymethyl)lysine in food systems by ultra performance liquid chromatography-mass spectrometry. Amino Acids.

[43] Wang J., Li Z., Pavase R.T., Lin H., Zou L., Wen J., Lv L. (2016). Advanced glycation endproducts in 35 types of seafood products consumed in eastern China. J. Ocean Univ. China.

[44] Bosch L., Sanz M.L., Montilla A., Alegría A., Farré R., del Castillo M.D. (2007). Simultaneous analysis of lysine, N^ε^-carboxymethyllysine and lysinoalanine from proteins. J. Chromatogr. B.

[45] Zhou Y., Lin Q., Jin C., Cheng L., Zheng X., Dai M., Zhang Y. (2015). Simultaneous analysis of N^ε^-(carboxymethyl)Lysine and N^ε^-(carboxyethyl)lysine in foods by ultra-performance liquid chromatography-mass spectrometry with derivatization by 9-fluorenylmethyl chloroformate. J. Food Sci..

[46] Niquet-Léridon C., Jacolot P., Niamba C.-N., Grossin N., Boulanger E., Tessier F.J. (2015). The rehabilitation of raw and brown butters by the measurement of two of the major Maillard products, *N^ε^*-carboxymethyl-lysine and 5-hydroxymethylfurfural, with validated chromatographic methods. Food Chem..

[47] Gómez-Ojeda A., Jaramillo-Ortíz S., Wrobel K., Wrobel K., Barbosa-Sabanero G., Luevano-Contreras C., de la Maza M.P., Uribarri J., Del Castillo M.D., Garay-Sevilla M.E. (2018). Comparative evaluation of three different ELISA assays and HPLC-ESI-ITMS/MS for the analysis of N^ε^-carboxymethyl lysine in food samples. Food Chem..

[48] Scheijen J.L., Clevers E., Engelen L., Dagnelie P.C., Brouns F., Stehouwer C.D., Schalkwijk C.G. (2016). Analysis of advanced glycation endproducts in selected food items by ultra-performance liquid chromatography tandem mass spectrometry: Presentation of a dietary AGE database. Food Chem..

[49] Poojary M.M., Zhang W., Greco I., De Gobba C., Olsen K., Lund M.N. (2020). Liquid chromatography quadrupole-Orbitrap mass spectrometry for the simultaneous analysis of advanced glycation end products and protein-derived cross-links in food and biological matrices. J. Chromatogr. A.

[50] Mavric E., Wittmann S., Barth G., Henle T. (2008). Identification and quantification of methylglyoxal as the dominant antibacterial constituent of Manuka (*Leptospermum scoparium*) honeys from New Zealand. Mol. Nutr. Food Res..

[51] Resmini P., Pellegrino L., Battelli G. (1990). Accurate quantification of furosine in milk and dairy products by a direct HPLC method. Ital. J. Food Sci..

[52] Schwietzke U., Malinowski J., Zerge K., Henle T. (2011). Quantification of Amadori products in cheese. Eur. Food Res. Technol..

[53] Troise A.D., Fiore A., Roviello G., Monti S.M., Fogliano V. (2015). Simultaneous quantification of amino acids and Amadori products in foods through ion-pairing liquid chromatography–high-resolution mass spectrometry. Amino Acids.

[54] Wellner A., Huettl C., Henle T. (2011). Formation of Maillard reaction products during heat treatment of carrots. J. Agric. Food Chem..

[55] Zhang G., Huang G., Xiao L., Mitchell A.E. (2011). Determination of advanced glycation endproducts by LC-MS/MS in raw and roasted almonds (*Prunus dulcis*). J. Agric. Food Chem..

[56] Henle T., Zehetner G., Klostermeyer H. (1995). Fast and sensitive determination of furosine. Z. Lebensm. Unters. Forsch..

[57] Henle T. (2008). Maillard Reaction of Proteins and Advanced Glycation End Products (AGEs) in Food. Process-Induced Food Toxicants.

[58] Drusch S., Faist V., Erbersdobler H.F. (1999). Determination of N^ϵ^-carboxymethyllysine in milk products by a modified reversed-phase HPLC method. Food Chem..

[59] Delatour T., Hegele J., Parisod V., Richoz J., Maurer S., Steven M., Buetler T. (2009). Analysis of advanced glycation endproducts in dairy products by isotope dilution liquid chromatography–electrospray tandem mass spectrometry. The particular case of carboxymethyllysine. J. Chromatogr. A.

[60] Henle T., Schwarzenbolz U., Klostermeyer H. (1997). Detection and quantification of pentosidine in foods. Z. Für Leb. Und-Forsch. A.

[61] Penndorf I., Biedermann D., Maurer S.V., Henle T. (2007). Studies on N-Terminal Glycation of Peptides in Hypoallergenic Infant Formulas:  Quantification of α-*N*-(2-Furoylmethyl) Amino Acids. J. Agric. Food Chem..

[62] Troise A.D., Dathan N.A., Fiore A., Roviello G., Di Fiore A., Caira S., Cuollo M., De Simone G., Fogliano V., Monti S.M. (2014). Faox enzymes inhibited Maillard reaction development during storage both in protein glucose model system and low lactose UHT milk. Amino Acids.

[63] Troise A.D., Fiore A., Colantuono A., Kokkinidou S., Peterson D.G., Fogliano V. (2014). Effect of Olive Mill Wastewater Phenol Compounds on Reactive Carbonyl Species and Maillard Reaction End-Products in Ultrahigh-Temperature-Treated Milk. J. Agric. Food Chem..

[64] Singapore Heart Foundation Triglycerides and Cholesterol Explained. https://www.myheart.org.sg/heart-news/triglycerides-vs-cholesterol/.

[65] Singapore Heart Foundation Understanding Blood Pressure Readings. https://www.myheart.org.sg/heart-news/blood-pressure-reading/.

[66] HealthHub Monitoring Blood Sugar for Exercise. https://www.healthhub.sg/programmes/diabetes-hub/monitoring-blood-sugar-for-exercise.

[67] Van Puyvelde K., Mets T., Njemini R., Beyer I., Bautmans I. (2014). Effect of advanced glycation end product intake on inflammation and aging: A systematic review. Nutr. Rev..

[68] Di Pino A., Currenti W., Urbano F., Scicali R., Piro S., Purrello F., Rabuazzo A. (2017). High intake of dietary advanced glycation end-products is associated with increased arterial stiffness and inflammation in subjects with type 2 diabetes. Nutr. Metab. Cardiovasc. Dis..

[69] Kim Y., Keogh J.B., Deo P., Clifton P.M. (2020). Differential effects of dietary patterns on advanced glycation end products: A randomized crossover study. Nutrients.

[70] Demirci B.G., Tutal E., Eminsoy I.O., Kulah E., Sezer S. (2019). Dietary fiber intake: Its relation with glycation end products and arterial stiffness in end-stage renal disease patients. J. Ren. Nutr..

[71] Yao D., Brownlee M. (2010). Hyperglycemia-induced reactive oxygen species increase expression of the receptor for advanced glycation end products (RAGE) and RAGE ligands. Diabetes.

[72] Slavin J. (2004). Whole grains and human health. Nutr. Res. Rev..

[73] Pletsch E.A. (2018). Investigating the Purported Slow Transit and Starch Digestion of Whole Grain Foods. Ph.D. Thesis.

[74] Rayaprolu S., Hettiarachchy N., Horax R., Satchithanandam E., Chen P., Mauromoustakos A. (2015). Amino acid profiles of 44 soybean lines and ACE-I inhibitory activities of peptide fractions from selected lines. J. Am. Oil Chem. Soc..

[75] Boirie Y., Dangin M., Gachon P., Vasson M.-P., Maubois J.-L., Beaufrère B. (1997). Slow and fast dietary proteins differently modulate postprandial protein accretion. Proc. Natl. Acad. Sci. USA.

[76] Kellow N.J., Coughlan M.T., Reid C.M. (2018). Association between habitual dietary and lifestyle behaviours and skin autofluorescence (SAF), a marker of tissue accumulation of advanced glycation endproducts (AGEs), in healthy adults. Eur. J. Nutr..

[77] Wang L., Jiang Y., Zhao C. (2024). The effects of advanced glycation end-products on skin and potential anti-glycation strategies. Exp. Dermatol..

[78] Verzijl N., DeGroot J., Thorpe S.R., Bank R.A., Shaw J.N., Lyons T.J., Bijlsma J.W., Lafeber F.P., Baynes J.W., TeKoppele J.M. (2000). Effect of collagen turnover on the accumulation of advanced glycation end products. J. Biol. Chem..

[79] Muir R., Forbes S., Birch D.J., Vyshemirsky V., Rolinski O.J. (2021). Collagen glycation detected by its intrinsic fluorescence. J. Phys. Chem. B.

[80] Atzeni I.M., Boersema J., Pas H.H., Diercks G.F., Scheijen J.L., Schalkwijk C.G., Mulder D.J., van der Zee P., Smit A.J. (2020). Is skin autofluorescence (SAF) representative of dermal advanced glycation endproducts (AGEs) in dark skin? A pilot study. Heliyon.

[81] Beisswenger P.J., Howell S., Mackenzie T., Corstjens H., Muizzuddin N., Matsui M.S. (2012). Two fluorescent wavelengths, 440_ex_/520_em_ nm and 370_ex_/440_em_ nm, reflect advanced glycation and oxidation end products in human skin without diabetes. Diabetes Technol. Ther..

[82] Förster A., Kühne Y., Henle T.O. (2005). Studies on absorption and elimination of dietary maillard reaction products. Ann. N. Y. Acad. Sci..

